# YES, AND...ENHANCING DEMENTIA CARE COMPETENCY THROUGH IMPROVISATIONAL THEATRE TECHNIQUES

**DOI:** 10.1093/geroni/igac059.2744

**Published:** 2022-12-20

**Authors:** Candace Kemp, Jennifer Craft Morgan, Andrea Hill, Emerald Pullon, Elisabeth Burgess, Molly Perkins, Alexis Bender

**Affiliations:** Georgia State University, Atlanta, Georgia, United States; Georgia State University, Atlanta, Georgia, United States; Georgia State University, Atlanta, Georgia, United States; Georgia State University, Atlanta, Georgia, United States; Georgia State University, Atlanta, Georgia, United States; Emory University School of Medicine, Atlanta, Georgia, United States; Emory University, Atlanta, Georgia, United States

## Abstract

As the number of persons living with dementia escalates globally, innovations in clinical practice are needed to promote care interactions that lead to positive outcomes for care recipients and their care partners, including family, friends, direct care workers, and other care providers. Our ongoing NIH-funded study, focused on meaningful engagement and quality of life, points to the potential benefits of training care partners to use improvisational (improv) theatre skills in the context of dementia care. We present analysis of data derived from two one-year-long waves of qualitative data collection involving persons with dementia (n=59), their formal and informal care partners (n=165), and participant observation (over 12,000 hours) in six diverse assisted living communities. We identified a minority of care partners who, relative to others, had greater success engaging residents and eliciting positive responses. They also experienced less frustration during care encounters. In interactions with persons with dementia, these individuals routinely extended and accepted invitations to communicate and demonstrated an ability to be in the moment and meet people where they were. They accepted uncertainty about interactions and recognized the need to be flexible. These approaches parallel key improv tenets, which are teachable. We argue that improv training for care partners would enhance care competency and led to positive outcomes for care recipients and providers. Findings contribute to a small, but growing innovative body of literature from a variety of disciplines, including health care, that demonstrates the benefits of improv training in a myriad scenarios and settings, including in dementia care.

